# Effects of health education on HIV/AIDS related knowledge among first year university students in China

**DOI:** 10.4314/ahs.v20i4.10

**Published:** 2020-12

**Authors:** Yan Liu, Li Lu, Yuan Yuan Wang, Meredith R Wilkinson, Yan-Ming Ren, Chao-Cai Wang, Fa-Bin Zhang, Jie Gao, Shou Liu

**Affiliations:** 1 Department of Public Health, Medical College, Qinghai University, Xining, Qinghai, China; 2 Team IETO, Bordeaux Population Health Research Center, UMR U1219, INSERM, Université de Bordeaux, Bordeaux, France; 3 Department of Psychology, Faculty of Health and Life Sciences, De Montfort University, Leicester, UK; 4 Qinghai Center for Disease Prevention and control, Xining, Qinghai, China; 5 Department of Clinical Medicine, Qinghai Institute of Health Sciences, Xining, Qinghai, China

**Keywords:** HIV/AIDS knowledge, awareness, health education, university students, China

## Abstract

**Background:**

The number of new HIV infections has increased and implementation of school-based health education programs on AIDS have been advocated for a long time.

**Objective:**

This study aimed to explore the effectiveness of an intervention of HIV/AIDS on the knowledge of HIV/AIDS prevention and control among first year university students.

**Methods:**

An awareness questionnaire was adopted to assess awareness and knowledge of HIV/AIDS pre- and post-health education among first year university students in Qinghai, China. Independent sample t-test, chi-square test, and multiple logistic regression analyses were used.

**Results:**

A total of 2,165 and 2,062 first year university students were respectively recruited pre- and post- HIV/AIDS health education. The awareness rate increased significantly after the health education intervention (from 48.59%, 95%CI: 46.47%–50.72% to 76.24%, 95%CI: 74.35%–78.06%). Students from Hui and Tibetan ethnicities, and those holding prejudices against AIDS patients were less knowledgeable than their counterparts regarding HIV/AIDS related knowledge, whereas urban-dwellers and those with higher paternal education were positively associated with awareness of HIV/AIDS related knowledge (p <0.05).

**Conclusion:**

HIV/AIDS awareness among first year university students improved greatly after receiving an education intervention, which underscores its utility as part of the approaches of HIV/AIDS control and prevention.

## Introduction

The human immunodeficiency virus and the acquired immunodeficiency syndrome (HIV/AIDS) are global public health issues and the leading causes of years lived with disability (YLDs) in many countries, such as South Africa, Lesotho and Swaziland, etc.^1^. The World Health Organisation (WHO) reported that there were 37.9 million people living with HIV worldwide at the end of 2018^2^, and that people living with HIV/AIDS in China increased from 385,817 in 2012 to 789,617 in 2018^3^. There are multiple ways in which HIV can be contracted, such as through the sharing of bodily fluids which contain the virus, notably blood and semen, which could be prevented and avoided.

The number of new HIV infections has increased in recent years, and sexual transmission was responsible for 90% of the infections^3^. As a sexually active population, university students are at a high risk of contracting sexually-transmitted diseases (STDs), such as HIV/AIDS^4^,^5^. The figure of sexual behavior among university students varied greatly among studies and different regions. The lifetime prevalence of risky sexual behavior among college students was 40.8% in Ethiopia^6^, one year prevalence was 72.2% in Zambia 7. A survey including 19,123 Chinese college students found that only 24.8% of sexually active college students reported using a condom for every sexual encounter 8. The high-risk sexual behavior among college students with regards to STDs is a matter of concern in public health^9^,^10^. The discriminatory attitudes to individuals who have HIV were also frequently reported, HIV-related stigma within health contexts is a broad social phenomenon which exists in diverse social spheres 11.

Implementation of school-based health education programs on AIDS have been advocated for a long time^12^–^14^ and students also have claimed positive attitude on sexual health education 15,16. Previous intervention studies have found a positive correlation between pro-active health behaviour and fact based health education 17,18. Specifically, one systematic review revealed that educational interventions have been successful at reducing stigma 19. Previous studies have indicated that making people more aware of HIV/AIDS leads to more positive attitudes towards HIV/AIDS patients 20,21. In addition, individuals can engage in more preventive behaviours and reduce the risk of contracting HIV (more specifically, sexually transmitted diseases) by increasing knowledge 22,23. Qinghai province of China has diverse population composition (such as Han, Tibetan, Hui, Salar, etc.), cultures, religions and languages. For example, Tibetans all have belief in Tibetan Buddhism, religion beliefs could affect various aspects of adolescents' social and daily life and students' attitudes on sexual behaviors^24^,^25^. Besides, some first year university students came from other parts of China, the educational backgrounds could be different from those who have lived in Qinghai since birth. However, sexual behaviors and related issues and health programs among university students were under-researched in Qinghai 26.

Therefore, this study aimed to explore the awareness and knowledge regarding HIV/AIDS prevention and control among first year university students in Qinghai, its key associated factors and the effects of health education. Based on previous studies 27,28, we hypothesized that awareness and knowledge would be generally improved after health education.

## Methods

### Study design, subjects and data collection

A ‘pre-post’ intervention study was conducted among first year university students during their military training at Qinghai University, China. There are 12 universities and colleges in Qinghai Province, and Qinghai University is the only university in Qinghai province listed in the national “211 Project”. It was selected as the pilot institution in Qinghai to conduct the pilot HIV/AIDS prevention and controlling intervention program launched by China's Ministry of Education. Cluster sampling was used to collect data before and after the courses. The pre-established questionnaire was sent out to the students who would attend the health education lectures (approximately all first year students attended the lectures) and collected by trained investigators on 31^st^ August, 2016 prior to the health education intervention. The same questionnaire was then sent out after students taking a one-hour health education lecture, which was delivered by doctors and associate chief physicians from the Department of Infectious Diseases of Qinghai Center for Disease Prevention and Control, from 1^st^ to 4^th^ September. In order to specifically benefit both sexes, the health education lectures were given separately among male and female students in different classrooms. All students were informed about the principle of anonymity and voluntary nature of the survey. A total of 2,165 and 2,062 students were involved preand post- the health education course, respectively and included students from different ethnic groups. The study protocol was approved by The Ethic Committee of Medical College of Qinghai University.

### Assessment tools

Socio-demographic characteristics, such as age, gender, ethnic groups (Han/ Hui/ Tibetan/ others), where students lived for the longest time before they entered university (village, township/ county /city), single parent family (yes/no), paternal and maternal education level (primary school or below/junior/ senior middle school/ above senior middle school), relationship status (previously in a relationship/ in a relationship/ not in a relationship) and attitudes towards HIV/ AIDS patients (sympathetic and helpful/ indifferent /fearful and avoiding) were included in the questionnaire.

The “Awareness questionnaire of knowledge on HIV/AIDS prevention and control - young student version ” was used to assess the status of first year students' knowledge and awareness on the knowledge on HIV/AIDS prevention and control 19. It was developed and recommended by the Chinese Center for Disease Control and Prevention in 2016 and has been applied in other Chinese studies 29. The questionnaire contains eight questions with the response being “yes”, “no” or “have no idea”, and the respondent would be considered as “aware” if they answered at least 6 out of the 8 questions correctly, otherwise “unaware”. Table 2 presents the questions which were asked.

**Table 2:** Awareness regarding HIV/AIDS prevention among first year students pre- and post- health education

	Pre- health education (N=2,165)		Post- health education (N=2,062)			
				
	Correct cases	Awareness rate (%)	Correct cases	Awareness rate (%)	*X*^2^	*P*
Q* 1	1,372	63.37	1,584	76.82	90.82	<0.001
Q 2	646	29.84	1,159	56.21	300.14	<0.001
Q 3	1,519	70.16	1,758	85.26	138.12	<0.001
Q 4	1,575	72.75	1,699	82.40	56.29	<0.001
Q 5	1,328	61.34	1,666	80.80	193.49	<0.001
Q 6	1,434	66.24	1,757	85.21	205.48	<0.001
Q 7	1,807	83.46	1,931	93.65	107.04	<0.001
Q 8	1,321	61.02	1,489	72.21	59.40	<0.001
Total	1,052	48.59	1,572	76.24	342.87	<0.001

* Q 1: Is AIDS an incurable and serious infectious disease?

Q 2: At present, the epidemic of AIDS among young students in China is growing rapidly, and the main mode of transmission is male homosexuality, followed by heterosexuality. Is that correct?

Q 3: Can you judge whether a person is infected with AIDS through appearance?

Q 4: Will people be infected with AIDS through daily life and learning contact?

Q 5: Does insisting on the correct use of condoms reduce the risk of infection and spread of AIDS?

Q 6: Will using new drugs (such as methamphetamine, ecstasy, K powder etc.) increase the risk of AIDS infection?

Q 7: After a high-risk behavior (needle sharing, unsafe sex etc.), should people actively seek HIV testing and counseling?

Q 8: Are the rights and interests (such as marriage, employment or enrolment etc.) of HIV-infected persons protected by the country's laws?

## Statistical analysis

Distribution of continuous data was examined using the Shapiro-Wilk test. Categorical and continuous variables were presented as number (N) and frequencies (%), and mean and standard deviation (SD), respectively. Chi-square (X^2^) test and independent sample t-test were appropriately used to explore the independent correlations between socio-demographic characteristics and awareness rate of HIV/AIDS pre- and posthealth education. Logistic regression models were built by setting “aware or not” as the dependent variable and other variables as independent variables to explore their correlations with awareness pre- and post-health education. Data analyses was conducted using STATA version 12.0 (STATA Corporation, College Station, Texas, USA) at the significance level of 0.05 (two-tailed).

## Results

A total of 3,840 first year students at the Qinghai University were given the questionnaire, of whom 2,165 (response rate: 56.4%) and 2,062 (response rate: 53.7%) returned the questionnaires pre- and post- the health education course, respectively. Socio-demographic characteristics regarding to awareness of knowledge on HIV/AIDS prevention and control among first year university students pre- and post- health education are shown in Table 1. As shown in Table 2, awareness rate increased significantly after receiving health education for the whole prevalence (pre-health education: 48.59%, 95%CI: 46.47%–50.72%; post-health education: 76.24%, 95%CI: 74.35%–78.06%; p <0.001) and also for each question.

**Table 1:** Socio-demographic characteristics regarding to HIV/AIDS awareness among first year students pre- and post- health education

Variables		Pre- Health education (N=2,165)				t / X^2^ (p)	Post- Health education (N=2,062)				t / X^2^ (p)
		
		Unaware		Aware			Unaware		Aware		
		Mean	SD	Mean	SD		Mean	SD	Mean	SD	
Age		18.72	0.98	18.54	0.92	4.33 (**<0.001**)	18.70	0.88	18.55	0.95	3.09 (**0.002**)
		N	%	N	%		N	%	N	%	
Gender	Female	522	47.07	587	52.93	17.14 (**<0.001**)	259	23.85	827	76.15	0.0093 (**0.923**)
	Male	591	55.97	465	44.03		231	23.67	745	76.33	
Ethnic groups	Han	611	44.37	766	55.63	87.81 (**<0.001**)	261	20.14	1,035	79.86	31.87 (**<0.001**)
	Hui	165	55.56	132	44.44		80	27.78	208	72.22	
	Tibetan	199	69.34	88	30.66		90	35.43	164	64.57	
	Other ethnic groups	138	67.65	66	32.35		59	26.34	165	73.66	
Where students lived for the longest time	Village, township	808	58.98	562	41.02	98.99 (**<0.001**)	355	28.09	909	71.91	36.10 (**<0.001**)
	County	185	44.58	230	55.42		76	19.29	318	80.71	
	City	120	31.58	260	68.42		59	14.60	345	85.40	
Single parent family	Yes	99	48.29	106	51.71	0.88 (0.348)	47	25.27	139	74.73	0.256 (0.613)
	No	1,014	51.73	946	48.27		443	23.61	1,433	76.39	
Paternal education	Primary school or below	347	62.86	205	37.14	61.06 (**<0.001**)	146	28.40	368	71.60	35.82 (**<0.001**)
	Junior middle school	435	52.35	396	47.65		215	27.89	556	72.11	
	Senior middle school	191	46.47	220	53.53		74	17.58	347	82.42	
	Above Senior middle school	140	37.74	231	62.26		55	15.45	301	84.55	
Maternal education	Primary school or below	518	60.16	343	39.84	63.58 (**<0.001**)	230	27.71	600	72.29	19.83 (**<0.001**)
	Junior middle school	354	50.79	343	49.21		152	23.57	493	76.43	
	Senior middle school	137	43.49	178	56.51		67	21.41	246	78.59	
	Above Senior middle school	104	35.62	188	64.38		41	14.96	233	85.04	
Relationship status	Previously in a relationship	267	50.86	258	49.14	0.13 (0.939)	120	23.90	382	76.10	0.65 (0.723)
	In a relationship	147	51.04	141	48.96		75	25.51	219	74.49	
	Not in a relationship	699	51.70	653	48.30		295	23.30	971	76.70	
Attitudes on HIV/ AIDS patients	Sympathetic and want to help	684	46.44	789	53.56	55.89 (**<0.001**)	322	21.52	1,174	78.48	29.06 (**<0.001**)
	Indifferent	204	56.20	159	43.80		63	22.83	213	77.17	
	Fear and avoidance	225	68.39	104	31.61		105	36.21	185	63.79	
Total		1,113	51.41	1,052	48.59		490	23.76	1,572	76.24	

Bold values: P<0.05

The description of independent variables in the logistic regression models are shown in Supplementary Table 1. Prior to the health education, those who lived in county (OR=1.50, 95%CI: 1.16–1.94) or city (OR=2.32, 95%CI: 1.75–3.08) for the longest time and students who had the paternal education of being above junior middle school (P<0.05) had positive correlations with the awareness of knowledge on HIV/AIDS prevention and control. While being male freshmen (OR=0.67, 95%CI: 0.56–0.81), Tibetan (OR=0.43, 95%CI: 0.32–0.58) and other ethnic groups (OR=0.47, 95%CI: 0.34–0.65), and those who were indifferent to (OR=0.63, 95%CI: 0.50–0.81) and had the feeling of fear and avoidance (OR=0.41, 95%CI: 0.31–0.53) on HIV/AIDS patients were negatively associated with the awareness (Table 3).

**Table 3:** Multiple logistic analyses pre- and post- the health education

Variables		Pre- Health education (N=2,165)			Post- Health education (N=2,062)		
		
		Coefficients	*P*-value	OR (95% CI)	Coefficients	*P*-value	OR (95% CI)
Age		-0.001	0.981	0.999 (0.90,1.12)	-0.02	0.685	0.98 (0.87, 1.10)
Gender	Male	-0.40	**<0.001**	0.67 (0.56, 0.81)	0.01	0.918	1.01 (0.82, 1.25)
Ethnic groups	Hui	-0.24	0.083	0.79 (0.60, 1.03)	-0.34	**0.032**	0.71 (0.52, 0.97)
	Tibetan	-0.84	**<0.001**	0.43 (0.32, 0.58)	-0.62	**<0.001**	0.54 (0.39, 0.74)
	Others	-0.76	**<0.001**	0.47 (0.34, 0.65)	-0.26	0.132	0.77 (0.55, 1.08)
Where students lived for the longest time	County	0.41	**0.002**	1.50 (1.16, 1.94)	0.37	**0.019**	1.45 (1.06, 1.98)
	City	0.84	**<0.001**	2.32 (1.75, 3.08)	0.57	**0.002**	1.76 (1.24, 2.49)
Single parent family	No	-0.25	0.108	0.78 (0.57,1.06)	0.08	0.685	1.08 (0.75, 1.55)
Paternal education	Junior middle school	0.26	**0.042**	1.29 (1.01, 1.65)	-0.11	0.431	0.90 (0.68, 1.18)
	Senior middle school	0.35	**0.025**	1.42 (1.05,1.92 )	0.41	**0.026**	1.50 (1.05, 2.15)
	Above Senior middle school	0.41	**0.041**	1.51 (1.02, 2.24)	0.41	0.098	1.51 (0.93, 2.45)
Maternal education	Junior middle school	0.05	0.688	1.05 (0.83, 1.32)	-0.05	0.715	0.95 (0.73, 1.24)
	Senior middle school	0.07	0.674	1.07 (0.78, 1.47)	-0.26	0.169	0.77 (0.53, 1.12)
	Above Senior middle school	0.18	0.400	1.20 (0.79, 1.82)	-0.11	0.693	0.90 (0.52,1.54)
Relationship status	In a relationship	0.003	0.984	1.00 (0.74, 1.36)	-0.14	0.420	0.87 (0.62, 1.22)
	Not in a relationship	0.01	0.932	1.01 (0.81, 1.25)	-0.03	0.815	0.97 (0.75, 1.25)
Attitude on HIV/AIDS patients	Indifferent attitude	-0.46	**<0.001**	0.63 (0.50, 0.81)	-0.13	0.417	0.88 (0.64, 1.20)
	Fear and avoidance	-0.90	**<0.001**	0.41 (0.31, 0.53)	-0.66	**<0.001**	0.52 (0.39, 0.68)

Post-health education, those who lived in county (OR=1.45, 95%CI: 1.06–1.98) or city (OR=1.76, 95%CI: 1.24–2.49) for the longest time, and students who had paternal education of senior middle school (OR=1.50, 95%CI: 1.05–2.15) have positive relationships with the awareness. While being Hui (OR=0.71, 95%CI: 0.52–0.97) and Tibetan (OR=0.54, 95%CI: 0.39–0.74) and students who had the feeling of fear and avoidance (OR=0.52, 95%CI: 0.39–0.68) on HIV/AIDS patients were inversely correlated with the awareness (Table 3).

## Discussion

This preliminary study explored the awareness regarding HIV/AIDS prevention and control before and after a health education intervention among first year university students in China's Tibetan area. The awareness rate increased significantly from 48.59% to 79.24% after the students received education and within each of the eight questions (see Table 2) of the questionnaire. The figure after health education was higher than that in Pakistan^30^, Ethiopia (59.6%) 15, and in Beijing, China (27.7%) 31. The differences among studies are likely due to the use of various assessment tools, and cultural norms 32. The significantly positive effects of related health education on HIV/AIDS knowledge have also been frequently identified in previous studies among university students conducted in other countries or regions, such as in Indonesia^33^, the United States 34,35 and Turkey 37.

Post health education, Hui and Tibetan students were less likely to be aware of HIV/AIDS prevention and control and related knowledge compared to the Han students, which could be partly attributed to cultural factors, such as language barriers in getting access to HIV/AIDS-related education 26. Most Tibetan and Hui students are religious, which may delay the age of having sexual intercourse and decrease the propensity to engage in sexual intercourse 38. Female students were more knowledgeable than their male counterparts prior to the health education, which is consistent with some of the previously published studies from Malaysia^39^ and the United States 40, but is not in agreement with some other studies which showed males were more knowledgeable than female students 41,42. However, posthealth education, gender differences were not found in our study which is similar to a study in Ajman 43.

Students who had lived for the most part of their lives in a village were less likely to be aware of HIV/AIDS prevention and control related knowledge than those lived mostly in a county or city, which were identified both pre- and post- health education. Similarly, it has identified that urban participants scored higher on HIV/AIDS prevention-related knowledge compared to rural participants in studies conducted in Beijing and Nanjing of China 44, as well as in some other countries, such as Jamaica 45 and Malaysia.^39^ Education and health-related information and services are more accessible in the developed areas compared to the geographically vulnerable regions in China^46^,^47^. Accordingly, HIV/AIDS related education and prevention programs in China should be implemented based on the needs and features of specific areas 44.

We also found that students who had paternal education of senior middle school had positive relationships with the awareness on HIV/AIDS related knowledge, while we did not identify the role of maternal education. Previous studies have shown that parents with higher education levels were more knowledgeable in general communication and HIV/AIDS knowledge, and fathers had higher sexual related knowledge than mothers^48^, which could partly help to explain our findings. The moderating effects of paternal education level between the residence of longest duration and awareness is still unknown and should be explored in further studies.

Most of the students in our study showed a sympathetic attitude and wanted to help the HIV/AIDS patients especially after receiving the health education. Several studies have indicated that the students' attitudes on HIV/AIDS patients were positively associated with their awareness 49,50, which were confirmed in our study. A study conducted in an Irish university reported that nearly 50% of the students were willing to be involved in social volunteer work with HIV/AIDS patients 51. It also showed that students with higher education levels had an overall positive attitude on many aspects of HIV/AIDS 41,52.

Our study showed that the awareness rate increased significantly after the health intervention program, hence educational departments and university administrators should promote this health education program regarding the HIV/AIDS related knowledge (which was part of a pilot HIV/AIDS prevention and controlling program launched by the China's Ministry of Education), to help college students to engender positive attitudes towards safe sex and improve their reproductive health.

There were some limitations in our study. First, only first year students from one university rather than multiple centers were included, so the findings should be interpreted with caution when generalized to university students in other places. Second, students were not questioned regarding their sexual experience, which might have an influence on their HIV/AIDS awareness. In addition, since the health education lectures were not given in Tibetan language, their effects on the Tibetan students maybe diluted. Dissemination of health education in different languages should be one of the primary concerns for future studies. Lastly, we totally followed the principle of voluntarism, which could partly lead to the low response rates in this study.

This research opens multiple avenues for future study. The first is taking a longitudinal approach in which participants are followed up 1-month and 1 year after initial participation. In these studies one could examine their current sexual activities to see if the education has had an impact and also their attitudes towards HIV/AIDS. It would also be good to run this study with students at high school level (age 16–18) so that one could see if there is an impact of administering the education intervention at an earlier age. A final avenue of research could examine the link between religiosity, knowledge of HIV/AIDS and sexual behaviours.

## Conclusion

Awareness of knowledge on HIV/AIDS prevention and control among university first year students improved greatly after receiving health education lectures. Hui and Tibetan students, those who were raised in less developed areas and those who have negative attitudes on HIV/AIDS patients were less knowledgeable. Due to the importance of HIV/AIDS prevention and control and its impact, effective measures and programs should be taken by the government and education department, so as to set health education as a regular part of students' curriculum.
